# Genomic analysis of 63,220 tumors reveals insights into tumor uniqueness and targeted cancer immunotherapy strategies

**DOI:** 10.1186/s13073-017-0408-2

**Published:** 2017-02-24

**Authors:** Ryan J. Hartmaier, Jehad Charo, David Fabrizio, Michael E. Goldberg, Lee A. Albacker, William Pao, Juliann Chmielecki

**Affiliations:** 1 Foundation Medicine, Inc, 150 2nd St, Cambridge, MA 02141 USA; 2 Roche Pharma Research & Early Development, Basel, Switzerland

**Keywords:** Neoantigens, Cancer vaccines, Genomic profiling, Poly-epitope

## Abstract

**Background:**

The integration of genomics with immunotherapy has potential value for cancer vaccine development. Given the clinical successes of immune checkpoint modulators, interest in cancer vaccines as therapeutic options has been revived. Current data suggest that each tumor contains a unique set of mutations (mutanome), thus requiring the creation of individualized cancer vaccines. However, rigorous analysis of non-individualized cancer immunotherapy approaches across multiple cancer types and in the context of known driver alterations has yet to be reported. We therefore set out to determine the feasibility of a generalizable cancer vaccine strategy based on targeting multiple neoantigens in an HLA-A/B subtype-directed manner.

**Methods:**

A cancer gene-focused, hybrid capture-based genomic analysis was performed on 63,220 unique tumors. Neoantigens were predicted using a combined peptide processing and MHC-I binding prediction tool (IEDB) for all recurrent (>10 tumors) missense alterations and non-frameshift indels for the two most common HLA-A/B subtypes in North American/European populations.

**Results:**

Despite being overwhelmingly unique overall, many mutanomes (~45%) contain at least one mutation from a set of ten mutations chosen to maximize the number of unique tumors. This held true for tumors driven by *KRAS* G12C (n = 1799), *PIK3CA* E545K (n = 1713), or *EGFR* L858R (n = 478) alterations, which define distinct sample subsets. We therefore hypothesized that sets of carefully selected mutations/neoantigens may allow the development of broadly applicable semi-universal cancer vaccines. To test the feasibility of such an approach, antigen processing and MHC-I binding prediction was applied for HLA subtypes A*01:01/B*08:01 and A*02:01/B*44:02. In tumors with a specific HLA type, 0.7 and 2.5% harbored at least one of a set of ten neoantigens predicted to bind to each subtype, respectively. In comparison, *KRAS* G12C-driven tumors produced similar results (0.8 and 2.6% for each HLA subtype, respectively), indicating that neoantigen targets still remain highly diverse even within the context of major driver mutations.

**Conclusions:**

This “best case scenario” analysis of a large tumor set across multiple cancer types and in the context of driver alterations reveals that semi-universal, HLA-specific cancer vaccine strategies will be relevant to only a small subset of the general population. Similar analysis of whole exome/genome sequencing, although not currently feasible at scale in a clinical setting, will likely uncover further diversity.

**Electronic supplementary material:**

The online version of this article (doi:10.1186/s13073-017-0408-2) contains supplementary material, which is available to authorized users.

## Background

Targeted cancer immunotherapies rely on antigens either unique to or highly enriched on tumor cells. Historically, efforts initially focused on self- or fetal antigens commonly overexpressed in tumors, potentially offering broadly applicable, targeted immunotherapy options [1–6]. However, targeting self-antigens alone was not able to stimulate a therapeutic immune response and these efforts were largely met with failure [7–9]. In contrast, somatic mutations can produce neoantigens (i.e., non-self) generating a robust antigen-specific response but are difficult to identify and are not common across tumor types [10–12]. Thus, leveraging neoantigens therapeutically is extremely challenging.

Next-generation sequencing provides the ability to identify somatically acquired mutations that have the potential to generate neoantigens and has therefore revitalized interest in cancer vaccines as a potential therapeutic strategy [11]. However, broad sequencing efforts have also uncovered immense genetic diversity both across and within tumors [13]. The widespread inter-tumor heterogeneity seen by The Cancer Genome Atlas and others suggests individualized cancer immunotherapy strategies may be required for a subset of patients with cancer. Early studies integrating genomics with cancer vaccine development in solid tumors show that individualized vaccines based, in part, on MHC-I binding predictions can be generated to elicit an immune response [11]. Yet despite these successes, developing individualized therapies still remains highly technical and difficult to scale. Mutanome engineered RNA immunotherapy (MERIT) is an emerging technology that aims to create rapidly deployed, individualized, poly-neo-epitope mRNA vaccines [14]. A central hallmark of MERIT is the extensive CD4+ T cell response the authors found against the majority of nonsynonymous mutations in murine tumor models. This suggests that MHC-II neoantigens can be leveraged towards immunotherapies more readily than MHC-I neoantigens. However, utilizing MHC-II prediction algorithms is difficult in a prospective approach since they have suboptimal rates of false positives and false negatives [15, 16]. It remains to be seen if vaccines created against multiple neoantigens can be combined in a “semi-universal poly-neoantigen” vaccine strategy applicable in a “non-individualized” manner.

Using a set of cancer gene-focused genomic profiles from 63,220 unique tumors, we set out to characterize genetic uniqueness for assessing the tenability of non-individualized cancer vaccines. To provide a conservative estimate of neoantigen production, we employed a multi-step in silico prediction of peptide processing, export, and MHC-I binding in a human leukocyte antigen (HLA) subtype-specific manner. In a separate analysis, MHC-II binding predictions were also employed. These data indicate that semi-universal, poly-neoantigen cancer vaccines containing realistic numbers of characterized cancer-associated neoantigen targets (i.e., 10–100) will be relevant to only a small subset of the general population. Thus, successful broad implementation of neoantigen-based targeted cancer immunotherapy strategies will be highly dependent on integration of genomic profiling with individualized therapies.

## Methods

### Tumor samples and sequencing

Samples were submitted to a CLIA-certified, New York State-accredited, and CAP-accredited laboratory (Foundation Medicine, Cambridge, MA, USA) for next-generation sequencing-based genomic profiling. The pathologic diagnosis of each case was confirmed by review of hematoxylin and eosin stained slides and all samples that advanced to nucleic acid extraction contained a minimum of 20% tumor cells. The samples used in this study were not selected and represent “all comers” to Foundation Medicine genomic profiling. Samples were processed in one of two broad protocols generally defined by solid tumors or hematologic cancers as previously described [17, 18]. For convenience, a brief description is provided below.

For solid tumors, DNA was extracted from formalin-fixed paraffin-embedded (FFPE) 10-micron sections. Adaptor-ligated DNA underwent hybrid capture for all coding exons of 287 or 395 cancer-related genes plus select introns from 19 or 31 genes frequently rearranged in cancer.

For hematologic cancers, DNA and RNA were extracted from either peripheral blood or bone marrow aspirate. Adaptor-ligated DNA underwent hybrid capture for all coding exons of 405 cancer-related genes. cDNA libraries prepared from RNA underwent hybrid capture for 265 genes known to be rearranged in cancer.

Captured libraries were sequenced to a median exon coverage depth of >500× (DNA) or approximately three million unique reads (RNA) using Illumina sequencing, and resultant sequences were analyzed for base substitutions, small insertions and deletions (indels), copy number alterations (focal amplifications and homozygous deletions), and gene fusions/rearrangements, as previously described [18]. Frequent germline variants from the 1000 Genomes Project (dbSNP142) were removed. To maximize mutation-detection accuracy (sensitivity and specificity) in impure clinical specimens, the test was previously optimized and validated to detect base substitutions at a ≥5% mutant allele frequency (MAF), indels with a ≥10% MAF with ≥99% accuracy, and fusions occurring within baited introns/exons with >99% sensitivity [17]. Known confirmed somatic alterations deposited in the Catalog of Somatic Mutations in Cancer (COSMIC v62) are called at allele frequencies ≥1% [19]. Patients were not consented for raw data release. Therefore, associated raw sequence data are not shared. However, variants from a subset of the samples used in this analysis (>18,000) have been deposited in the Genomic Data Commons (accession number phs001179).

### Neoantigen prediction

All missense single nucleotide variants (SNVs) and non-frameshift indel variants occurring in at least ten tumor samples were used for neoantigen prediction. A cutoff of ten tumors represents only 0.016% (10/63,220) of the sample set. Since neoantigen prediction is dependent on HLA subtype and the most common HLA subtype population frequency in North Americans is ~10%, it is unlikely to find any shared neoantigens below this frequency. The flanking ±25 amino acids surrounding each missense SNV and non-frameshift indel variant were obtained similarly for both the wild type (WT) and variant.

Frameshift events were excluded since they are uncommonly shared across tumors (the most common frameshift alteration is found in ~0.5% of tumors). The potential for novel peptides is limited since most frameshift alterations (~50%) result in a stop codon within 15 amino acids (data not shown) and these will often result in transcript degradation prior to peptide translation. For these reasons, without direct validation of peptide MHC-I binding we felt the risk of false positive neoantigens outweighed the likelihood of shared frameshift variants producing neoantigens.

For MHC-I, an end-to-end peptide processing and MHC-I binding predictor (IEDB) [20] was used for both WT and variant peptide fragments (via the API; http://tools.iedb.org/main/tools-api/). This tool produces an overall antigen estimate by combining predictions for proteosomal processing (using “immuno” proteasome type), TAP transport, and MHC-I binding. For MHC-I binding, NetMHCpan was used with specific HLA-A/B subtypes. HLA-specific binding thresholds were utilized to dichotomize each peptide as an MHC-I binder or a non-binder, as described previously [21]. Finally, all variant peptide MHC-I binders were filtered against WT MHC-I binders. This enriched for predicted binders specific to the variant while also allowing for the inclusion of novel peptide fragments created, for example, by the disruption of peptide cleavage sites.

For MHC-II, an MHC-II binding prediction tool (IEDB) using the “consensus method” (as previously described [15, 16]) was used for both WT and variant peptides for the most common HLA-DRB, HLA-DQA, HLA-DQB, and HLA-DPA subtypes. Since binding thresholds for MHC-II are not well established, a “low affinity” and a “high affinity” threshold were used (IC50 values of 500 and 50 nm, respectively). To avoid false positives, MHC-II binding peptides were required to have a predicted IC50 binding affinity less than this threshold in both the SMM and NN methods within the “consensus method”. Similarly to MHC-I binders, mutant-specific MHC-II peptide binders were determined by filtering against all WT peptide MHC-II binders for each specific MHC-II HLA subtype.

All HLA subtype population frequencies were obtained via the Allele Frequency Net Database [22].

### HLA typing for neoantigen prediction

Since HLA loci are captured as part of the hybrid-capture panel, sequence-based HLA typing was possible. Neoantigen prediction using population-wide HLA assumptions was compared to tumor-derived HLA types in a subset of tumors. Specifically, tumor-derived HLA type neoantigen predictions were performed for a randomly selected set of 40 lung adenocarcinomas harboring a *KRAS* G12C alteration. Sequence-derived HLA-A/B/C typing was conducted by back-converting BAM files to fastq, then performing HLA realignment and typing using OptiType [23]. All variants within each tumor were then utilized with the corresponding tumor-derived HLA type for neoantigen prediction as described above.

## Results

### Tumor mutanomes are unique

**Fig. 1:**
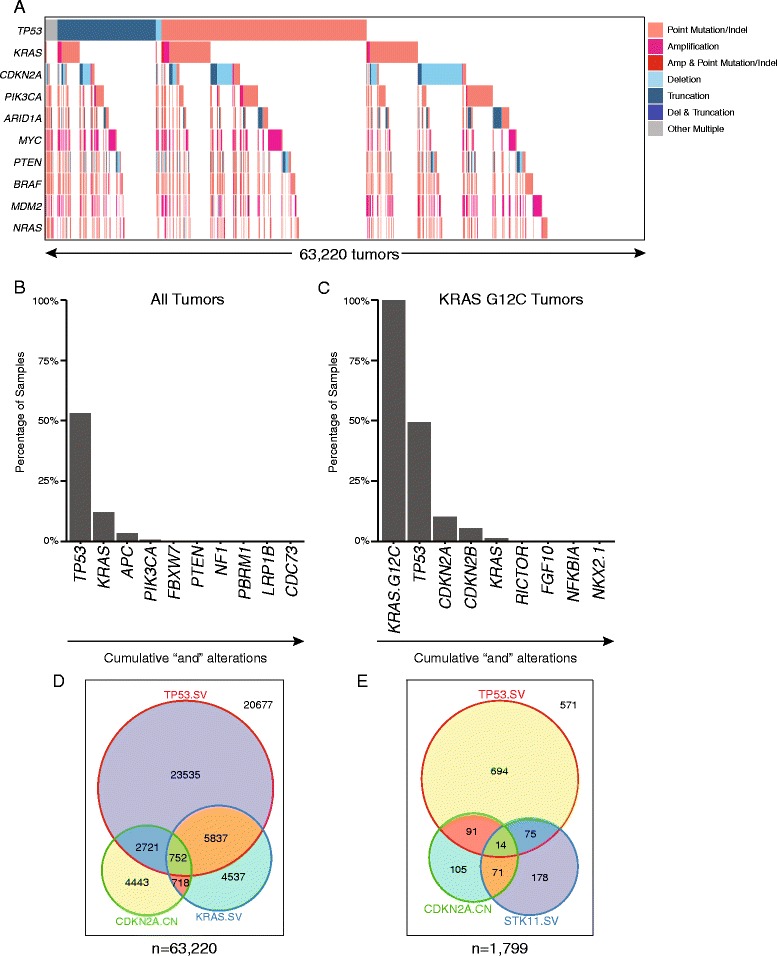
Tumor mutanomes are overwhelmingly unique. **a** The alteration classes in frequently mutated genes across 63,220 tumors. **b**, **c** Top cumulative “and” alterations (tumors which contain all alterations from *left* to *right*) for **b** all tumors or **c** *KRAS* G12C-driven tumors. **d**, **e** The overlap of the top three alteration types across **d** all tumors or **e** *KRAS* G12C-driven tumors

We first examined the set of genomic alterations from each tumor (mutanome) across all samples to understand the extent and context of tumor uniqueness. Uniqueness was defined by the set of alterations in a tumor in three ways: (1) at the gene level (i.e., *KRAS*); (2) at the variant type level (i.e., *KRAS* SNV, *KRAS* copy number, etc.); and (3) at the variant level (i.e., *KRAS* G12C). Inspection of this relatively narrow portion of the coding genome revealed that the majority of tumors contained unique mutanomes for “gene” to “variant” level uniqueness (range 72–95%). This was similar for subsets of tumors with known driver mutations, including *KRAS* G12C (78–93%) and *EGFR* L858R (77–95%). Non-unique mutanomes tended to have fewer alterations, sometimes containing only a single driver mutation. We thus examined whether a subset of mutanomes are shared across samples by identifying genes with alterations frequently co-occurring in a maximally cumulative manner (cumulative “and” alterations). A tile plot for the top ten genes across all 63,220 tumors revealed that although these genes are frequently mutated, few samples have more than two to three altered genes in common (Fig. 1a). For example, only ~5% of samples contain alterations in *TP53*, *KRAS*, and *APC* (Fig. 1b). *KRAS* G12C tumors show a similar pattern, albeit with distinct genes: *TP53*, *CDKN2A/B*, and secondary *KRAS* variants (Fig. 1c). A breakdown of tumor types within these groups is shown in Additional file 1: Figure S1. *EGFR* L858R lung adenocarcinomas similarly share few alterations between tumors. Variant type level uniqueness for the top three alterations further establish the minimal overlap between tumors (Fig. 1d, e). Together, these data suggest that tumors have remarkably few shared alterations with other tumors, even in the context of major driver alterations and in specific disease types.

### Small sets of alterations are found across many tumors

**Fig. 2:**
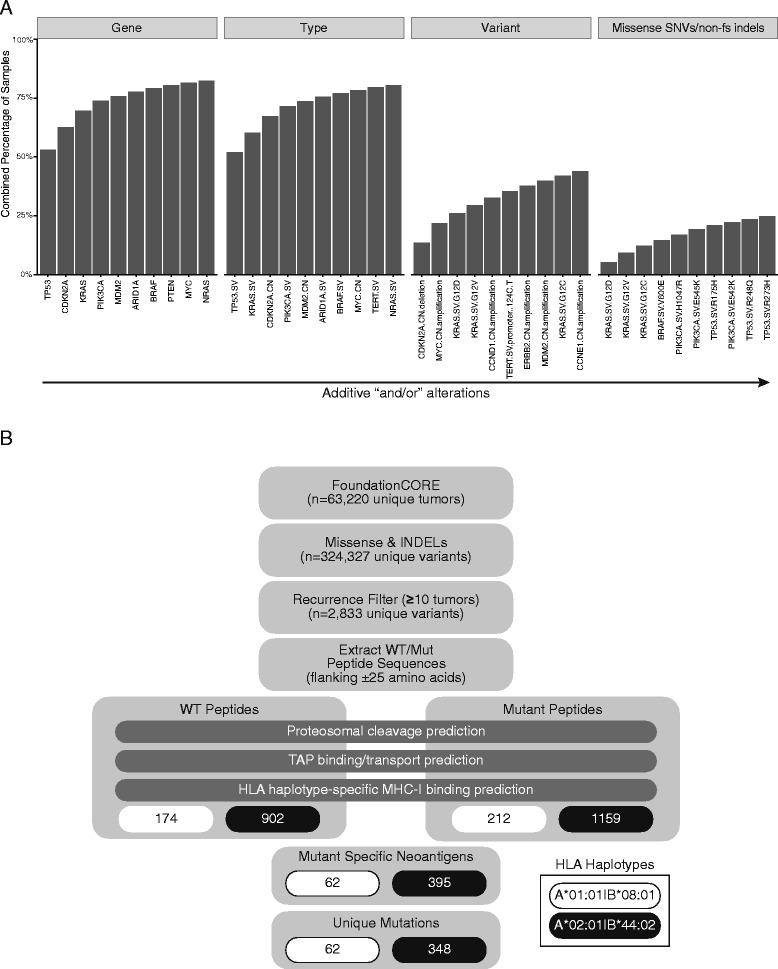
Sets of alterations shared across many tumors. **a** Top additive “and/or” alterations were determined by maximizing the number of unique tumors containing one or more alterations (from *left* to *right*; i.e., tumors with gene 1 and/or gene 2, etc.). Overlap across variants was determined by four broad categories (*Gene*, *Type*, *Variant*, *Missense SNVs/non-frameshift (fs) indels*). **b** Neoantigen prediction strategy incorporating the number of peptides processed and predicted to bind to specific HLA subtypes

We next asked whether at least one alteration in a relatively small set of alterations (picked in a way to maximize unique tumors) can be found across many tumors. This has implications for cancer immunotherapy development since many antigens could be targeted (even if they are not all present in a given tumor), thereby making a single cancer vaccine broadly applicable [24]. Alterations maximizing the number of unique tumors with at least one alteration were therefore identified (additive “and/or” alterations). At the level of “gene uniqueness”, across the 63,220 tumors, over 75% possess an alteration in at least one of ten genes (Fig. 2a, “Gene”). Although this dropped precipitously for missense SNVs, ~25% of tumors contain at least one of a set of only ten variants (Fig. 2a, “Missense SNVs”). These data suggest the possibility of identifying relatively small sets of variants for the creation of broadly applicable, non-individualized cancer immunotherapies. To fully evaluate the tenability of this approach, rigorous neoantigen predictions were employed.

### Identification of neoantigens

Antigen presentation begins with peptide cleavage and transport to the endoplasmic reticulum and concludes with binding to MHC-I molecules for presentation. Each of these steps involves enzymes or molecules with non-random peptide preferences. HLA genes are highly polymorphic, resulting in vastly different peptide binding affinities. In fact, analysis of all unique missense SNVs across the 12 most common North American/European HLA-A/B subtypes revealed that MHC-I binding is highly dependent on HLA subtype (Additional file 1: Figure S2) and most presented antigens are restricted to a single HLA type. Thus, neoantigen prediction must be done in an HLA-specific manner.

With this in mind, an end-to-end neoantigen prediction pipeline combining peptide processing, TAP transport, and MHC-I binding [20] was implemented for the two most common North American/European HLA-A/B subtypes, A*01:01|B*08:01 and A*02:01|B*44:02 (Fig. 2b). Epitope prediction was performed for both WT and mutant peptide sequences from all coding missense variants and non-frameshift indels found in ≥10 tumors. Mutant specific antigens (neoantigens) were identified by filtering against predicted WT epitopes. For the two HLA-A/B subtypes, 62 and 348 mutant-specific MHC-I epitopes were predicted to be generated as a consequence of 62 and 395 alterations, respectively. These data indicate that in this dataset, 2% (62/2833) and 12% (348/2833) of recurrent missense SNVs and non-frameshift indels are predicted to produce a unique neoantigen for A*01:01|B*08:01 and A*02:01|B*44:02 subtypes, respectively.

### Identification of shared neoantigens for non-individualized targeted cancer immunotherapies

**Fig. 3:**
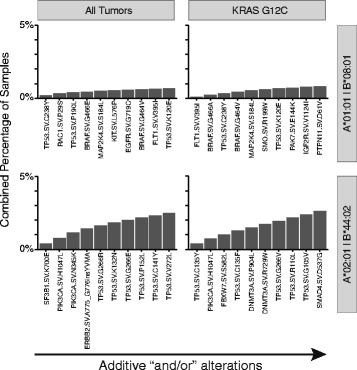
Applicability of poly-neoantigen, non-individualized targeted cancer immunotherapies using peptide processing, and MHC-I binding predictions. Top additive “and/or” alterations predicted to produce an MHC-I neoantigen are shown for all tumors (*left*) and KRAS G12C-driven tumors (*right*) for two common HLA-A/B subtypes, A*01:01/B*08:01 (*top*) and A*02:01/B*44:02 (*bottom*)

To examine the applicability of non-individualized poly-neoantigen cancer immunotherapies, sets of neoantigen-producing alterations maximizing the number of unique tumors were determined (additive “and/or” alterations). This was conducted across all tumors focusing on ten predicted neoantigen producers and in the context of a major driver alteration (*KRAS* G12C) for two major HLA-A/B subtypes (A*01:01|B*08:01 and A*02:01|B*44:02). Since these neoantigens have not been empirically validated and the tested HLA-A/B subtypes are common, this represents a “best-case scenario” for the generalizability of this approach. Across all tumors with a specific HLA subtype, only 0.7–2.5% of tumors contain one or more alteration from a set of ten predicted neoantigen producers (Fig. 3). Taking into account HLA subtype population frequencies (A*01:01|B*08:01 = 12.6%; A*02:01|B*44:02 = 10.8%), this translates to less than ~0.3% of the general population (A*01:01|B*08:01 = 0.7% × 12.6% = 0.09%; A*02:01|B*44:02 = 2.5% × 10.8% = 0.31%). Similar results were observed for *KRAS* G12C-driven tumors. Furthermore, including all variants producing neoantigens across all tumors only slightly expanded these numbers for each HLA-A/B subtype (1.3 and 9.3%) and for the general population (0.2 and 1.0%). These data indicate that few tumors share variants that lead to HLA-specific neoantigens and that any non-individualized semi-universal cancer immunotherapy strategy will only be applicable to an extremely limited portion of the population.

These analyses rely on assumed HLA type based on general population frequencies. To test whether the results hold true for patient-specific HLA types, HLA types were determined for a set of 40 lung adenocarcinomas with a *KRAS* G12C alteration. Neoantigens were then identified based on the specific variants identified and tumor derived HLA type. The only neoantigen identified in more than one tumor was *KRAS* G12C, which produces a neoantigen for a single HLA type (HLA-A*11:01; US population frequency = 10.4%). Of the 40 *KRAS* G12C tumors examined for tumor-derived HLA neoantigen prediction, eight were HLA-A*11:01. This did not significantly differ from that expected utilizing population-based HLA frequencies (*p* = 0.35 by Fisher’s exact test). Thus, tumor-derived HLA type neoantigen prediction supports the more general analysis that shared neoantigens are rare.

To examine the impact of MHC-II-presented neoantigens, a similar approach was undertaken using MHC-II peptide binding predictions (Additional file 1: Figure S3). However, MHC-II peptide binding has much more uncertainty than MHC-I predictions, especially with respect to binding thresholds. Using a “high” and a “low” binding affinity threshold (50 and 500 nM, respectively) across two “consensus” prediction algorithms produced a large variation in the number of predicted neoantigens. Thus, without further refinement or validation of predicted MHC-II targets, the utility of this form of neoantigen presentation remains unclear.

## Discussion

Our data reveal that inter-individual tumor genomic heterogeneity is extensive, even in the context of known driver mutations, and suggest that targeted cancer vaccines may need to be generated specifically for each patient. However, it is currently not feasible to scale these technologies to large populations. We thus sought to explore the tenability of non-individualized targeted immunotherapies by focusing on poly-neoantigen targeting strategies. In summary, sets of neoantigens were identified in an HLA subtype-specific manner that could be used to generate cancer vaccines applicable to subsets of the cancer population. However, in a “best-case scenario” analysis, each set of neoantigens would be relevant to less than ~0.3% of the population. Surprisingly, this was not impacted by the presence of a major driver mutation or by examining specific diseases, and maximizing the number of neoantigens per set to >100 had only a modest impact. Although this is already a small proportion of tumors, it is likely a substantial overestimation for the following reasons.

First, our analysis was based solely on alterations identified from DNA sequencing. It is likely that some of the alterations do not create neoantigens because the gene (or variant allele) is not transcribed/translated. Second, neoantigen prediction will produce some false positives that are impossible to identify without direct validation. Third, clonality, which has been shown to influence the neoantigen immune response [25], was not incorporated into this analysis. Fourth, selective pressures may reduce the number of neoantigens present in a given HLA subtype. Fifth, sequencing was done without matched normal samples. Although variants were heavily filtered for known germline polymorphisms, it is possible some of the neoantigens identified are rare germline events not appropriate for targeted immunotherapies. Sixth, the bulk of the analysis relied on assumed HLA frequencies rather than measured HLA types. Incorporation of measured HLA types into this analysis would likely further reduce the fraction of shared neoantigens. In support of this, genomically determined HLA type neoantigen prediction was performed across 40 tumors with similar results. This raises the possibility of targeting *KRAS* G12C in HLA-A*11:01 patients. However, overall these data suggest limited applicability for non-individualized targeted immunotherapies.

An important limitation of this analysis is that it is based on targeted sequencing data. We cannot exclude the possibility that critical variants producing neoantigens across many tumors exist in un-sequenced regions of the exome. Further, since it has been shown that neoantigens are less likely to occur in cancer-associated genes [26], the rate of neoantigens across the remainder of the exome could be significantly higher than we observed. However, variants in non-cancer-associated genes are unlikely to be recurrent across tumors. Our inclusion of all benign and uncharacterized variants helped reduce the impact of biological selective pressure on neoantigen identification. Further, exome sequencing in lung adenocarcinoma [27] revealed few shared mutations predicted to produce neoantigens based on patient-specific HLA type MHC-I binding predictions. Importantly, our analysis implemented a refined definition of “shared neoantigens” based on unique peptides, not mutations, which likely further reduced the number of shared neoantigens. This is important for cancer vaccine development since a given mutation can produce many distinct peptides, each with their own MHC-I affinities. Thus, the main conclusions of this study are unlikely to be significantly altered by the reliance on targeted sequencing data.

Another important limitation is the exclusion of frameshift alterations. These alterations were excluded because of the high likelihood for early stop codons and subsequent transcript degradation by nonsense-mediated degradation. Although these alterations have the potential to create novel peptides for neoantigen targeting, the risk of false positive neoantigens was deemed too great without direct validation of peptide MHC-I binding. Thus, we cannot exclude the possibility that our analysis missed bone fide shared neoantigens produced from frameshift alterations.

Our analysis was also focused on predicted MHC-I antigen binding due to a wide degree of uncertainty in predicting the binding threshold of MHC-II peptides. Although MHC-II plays an important role in antigen presentation, in silico MHC-II antigen prediction is currently not as reliable to inform immunotherapy strategies. However, MHC-II-presented peptides have the potential to produce a large number of neoantigens and should continue to be examined as identification efforts improve.

## Conclusions

It is possible to identify a set of alterations shared across patient tumors for the production of a non-individualized, poly-neoantigen cancer vaccine in an HLA subtype-specific manner. However, with current neoantigen prediction methodologies, this approach will be applicable to only a small proportion of the population.

## Additional file

Additional file 1: Supplementary figures S1-S3. **Figure S1.** Tumor type breakdown across the dataset and tumors with the *KRAS* G12C mutation. **Figure S2.** Number of common HLA subtypes a given peptide is predicted to bind. **Figure S3.** Examination of MHC-II predicted shared neoantigens. (PDF 115 kb)

## Abbreviations

FFPE: Formalin-fixed paraffin-embedded
HLA: Human leukocyte antigen
IEDB: Immune Epitope Database and Analysis Resource
MERIT: Mutanome Engineered RNA Immunotherapy
MHC: Major histocompatibility complex
SNV: Single nucleotide variant
