# Supplementary material for: Genomic analysis of 63,220 tumors reveals insights into tumor uniqueness and targeted cancer immunotherapy strategies
Source: Genome Med. 2017 Feb 24;9:16. doi: 10.1186/s13073-017-0408-2 (PMC5324279; doi:10.1186/s13073-017-0408-2)
Supplement: Additional file 1: — Supplementary figures S1-S3. Figure S1. Tumor type breakdown across the dataset and tumors with the KRAS G12C mutation. Figure S2. Number of common HLA subtypes a given peptide is predicted to bind. Figure S3. Examination of MHC-II predicted shared neoantigens. (PDF 115 kb) [file 13073_2017_408_MOESM1_ESM.pdf]

ADDITIONAL FILE 1: Supplementary Figures

Genomic analysis of 63,220 tumors reveals insights into tumor uniqueness and targeted cancer immunotherapy strategies

Ryan J. Hartmaier[1,*], Jehad Charo[2], David Fabrizio[1], Michael E. Goldberg[1], Lee A. Albacker[1], William Pao[2], Juliann Chmielecki[1]

1. Foundation Medicine, Inc., Cambridge MA, USA
2. Roche Pharma Research & Early Development, Basel, Switzerland

SUPPLEMENTAL FIGURES

Figure S1

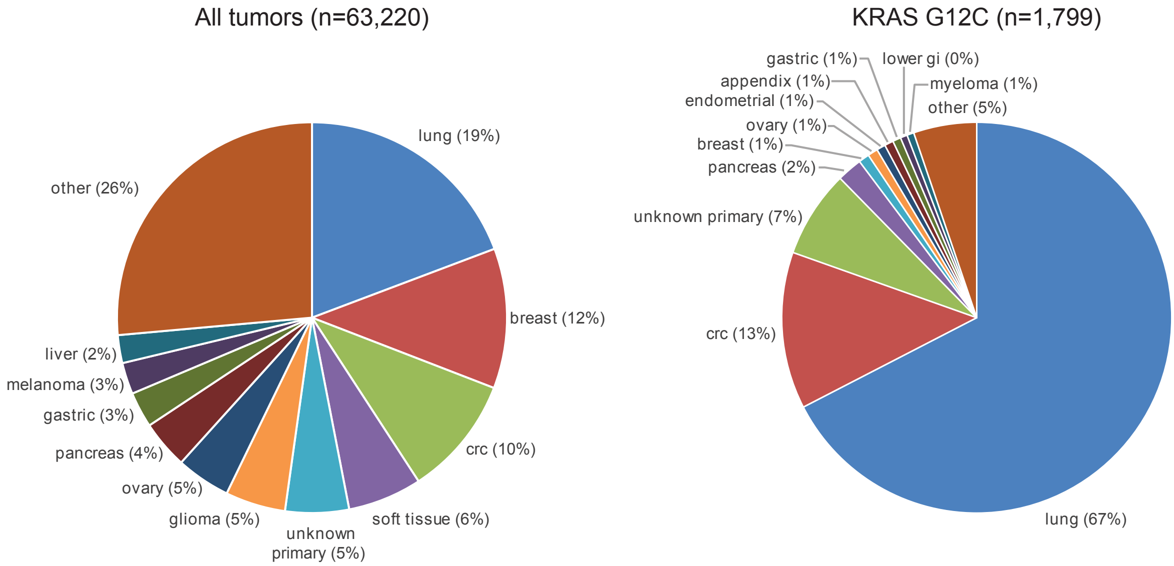

Figure S1: Tumor type breakdown.

Pie charts show the breakdown of tumor types across all tumors (n=63,220) and within the *KRAS* G12C containing tumors (n=1,799). The top 11 tumor types are shown with all others being categorized as 'other.'

Figure S2

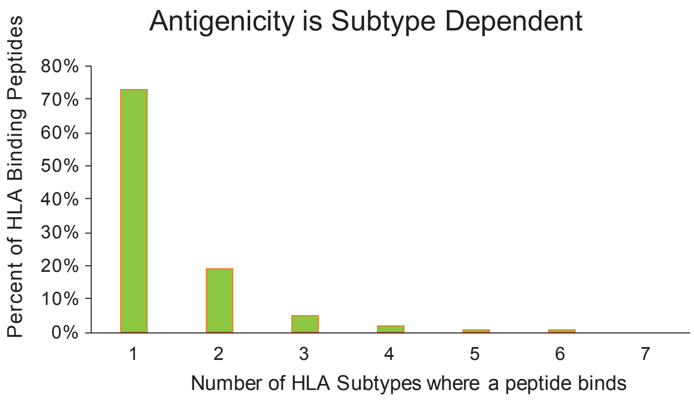

Figure S2: Antigenicity is HLA-subtype dependent

MHC-I binders were determined across 12 common HLA-subtypes. The total number of HLA-subtypes predicted to bind a given peptide were counted. Most peptides bind to a single HLA-subtype.

Figure S3

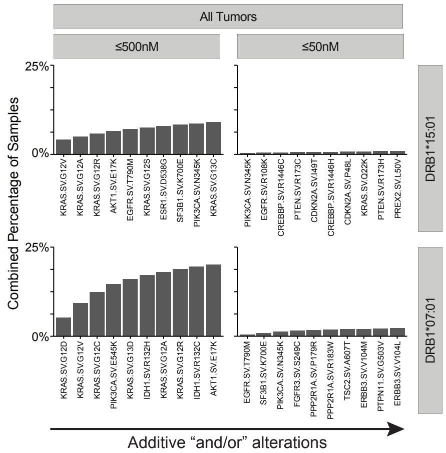

Figure S3: Applicability of poly-neoantigen, non-individualized targeted cancer immunotherapies MHC-II binding predictions

Top additive “and/or” alterations predicted to produce an MHC-II neoantigen are shown for all tumors using ‘low’ and ‘high’ affinity thresholds (500nM and 50nM). This was done for two common HLA-DRB1 subtypes (DRB1*15:01 and DRB1*07:01).
